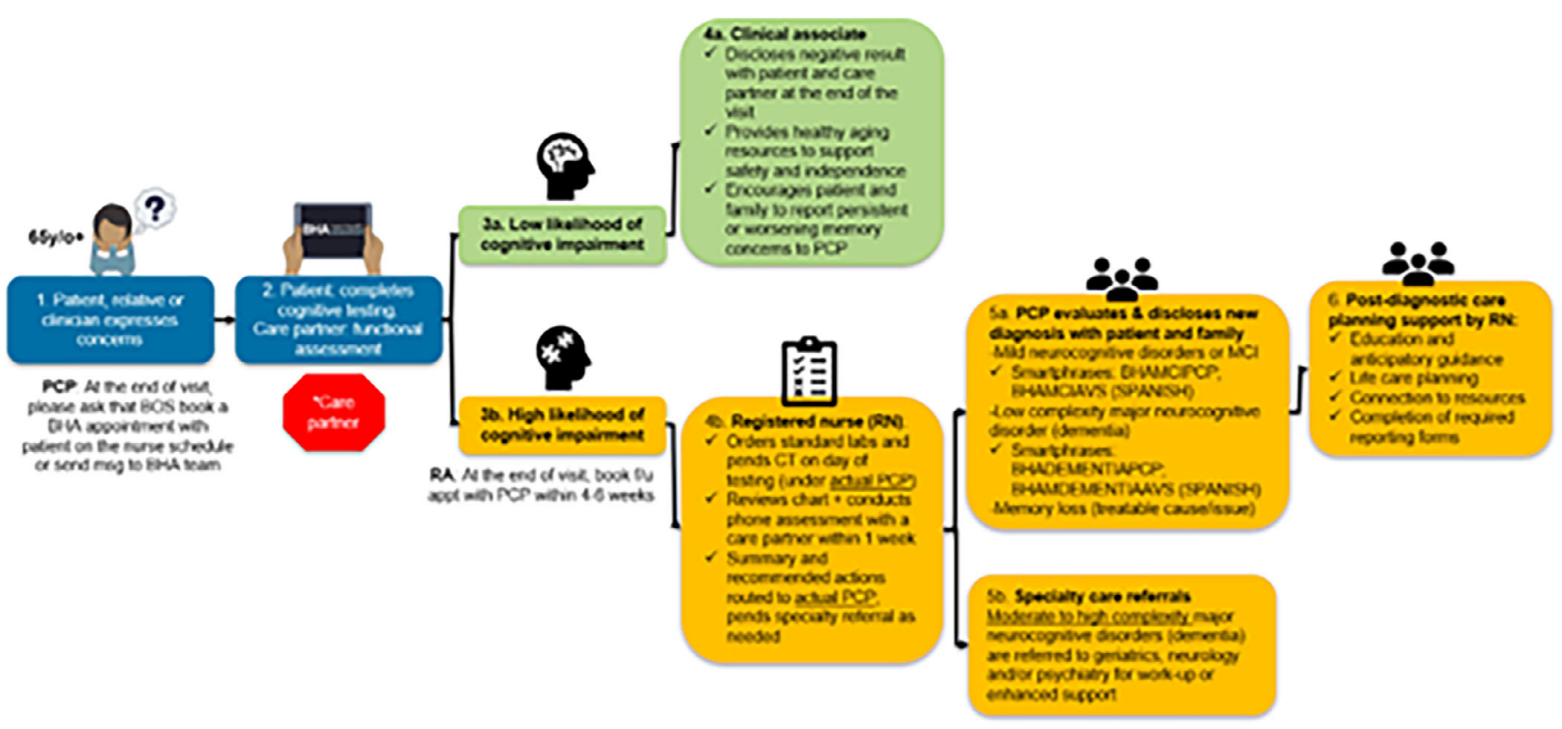# Early Learnings from a Stakeholder‐Informed Pragmatic Trial of the TabCAT‐Brain Health Assessment for the Detection of Cognitive Impairment in Primary Care

**DOI:** 10.1002/alz70858_102199

**Published:** 2025-12-25

**Authors:** Huong Q Nguyen, Annette Langer‐Gould, Eric A Lee, Alissa Bernstein Sideman, Mayra Macias, Soo Borson, Ernest Shen, Elena Tsoy, Katherine L. Possin

**Affiliations:** ^1^ Kaiser Permanente Southern California, Pasadena, CA, USA; ^2^ Kaiser Permanente Southern California, Los Angeles, CA, USA; ^3^ Global Brain Health Institute, San Francisco, CA, USA; ^4^ University of California, San Francisco, San Francisco, CA, USA; ^5^ Keck USC School of Medicine, Los Angeles, CA, USA; ^6^ BOLD Center on Early Detection of Dementia, New York, NY, USA; ^7^ Memory and Aging Center, University of California San Francisco, San Francisco, CA, USA; ^8^ University of California San Francisco, San Francisco, CA, USA; ^9^ UCSF Memory and Aging Center, San Francisco, CA, USA; ^10^ Memory and Aging Center, San Francisco, CA, USA; ^11^ Memory and Aging Center, Weill Institute for Neurosciences, University of California, San Francisco, San Francisco, CA, USA; ^12^ Global Brain Health Institute (GBHI), University of California San Francisco (UCSF); & Trinity College Dublin, San Francisco, CA, USA

## Abstract

Implementing scalable and sustainable, evidence‐informed diagnostic care pathways to improve earlier detection of cognitive impairment (CI) in primary care requires extensive engagement with front‐line clinicians, physician and administrative leaders and careful coordination with intersecting specialties. In preparation for a pragmatic, cluster, randomized trial with 26 primary care clinics in an integrated health system, we conducted pre‐implementation stakeholder engagement meetings to adapt core elements of practice support: 1) provider education and training; 2) digital tools to assess cognitive function (TabCAT‐Brain Health Assessment) and support clinician decision making and documentation; and 3) registered nurse (RN) support during the work‐up and post‐diagnosis periods for primary care physicians (PCPs), patients, and families. We used thematic analysis of meeting transcripts to guide intervention adaptations. The overarching theme from these meetings was that PCPs want to provide the best brain health care possible within the constraints of their limited time and gaps in expertise. Patients with memory concerns complete the TabCAT‐BHA with a nonclinical staff who provide results to RNs. For individuals found to have a high likelihood of CI, the RN assesses the patient's functional status and behavior with a care partner, reviews the medical record, and triangulates findings with cognitive test results to arrive at a preliminary classification of mild cognitive impairment (MCI) or dementia, and orders lab tests and imaging on behalf of the PCP. The RN then routes a summary to the patient's PCP with scripted guidance on disclosing a new diagnosis with the patient/family, or, for complex patients, pends a specialty referral for PCP approval. Once the PCP has disclosed the initial diagnosis of MCI or dementia, the RN then provides initial care planning and navigation with the patient/family. A total of 740 Tab‐CAT BHAs have been completed since March 2024, with 80% of patients (54% Spanish speakers) having a classification of mild or major neurocognitive disorder. PCPs’ reception of the program has been overwhelmingly positive, with 85% reporting that they are very to extremely likely to recommend the TabCAT‐BHA care pathway to their peers. Practice supports must be tailored to local contexts to achieve high acceptability and sustainability.